# A Widespread Chromosomal Inversion Polymorphism Contributes to a Major Life-History Transition, Local Adaptation, and Reproductive Isolation

**DOI:** 10.1371/journal.pbio.1000500

**Published:** 2010-09-28

**Authors:** David B. Lowry, John H. Willis

**Affiliations:** 1 University Program in Genetics and Genomics, Duke University Medical Center, Durham, North Carolina, United States of America; 2 Department of Biology, Duke University, Durham, North Carolina, United States of America; University of Edinburgh, United Kingdom

## Abstract

The role of chromosomal inversions in adaptation and speciation is controversial. Historically, inversions were thought to contribute to these processes either by directly causing hybrid sterility or by facilitating the maintenance of co-adapted gene complexes. Because inversions suppress recombination when heterozygous, a recently proposed local adaptation mechanism predicts that they will spread if they capture alleles at multiple loci involved in divergent adaptation to contrasting environments. Many empirical studies have found inversion polymorphisms linked to putatively adaptive phenotypes or distributed along environmental clines. However, direct involvement of an inversion in local adaptation and consequent ecological reproductive isolation has not to our knowledge been demonstrated in nature. In this study, we discovered that a chromosomal inversion polymorphism is geographically widespread, and we test the extent to which it contributes to adaptation and reproductive isolation under natural field conditions. Replicated crosses between the prezygotically reproductively isolated annual and perennial ecotypes of the yellow monkeyflower, *Mimulus guttatus*, revealed that alternative chromosomal inversion arrangements are associated with life-history divergence over thousands of kilometers across North America. The inversion polymorphism affected adaptive flowering time divergence and other morphological traits in all replicated crosses between four pairs of annual and perennial populations. To determine if the inversion contributes to adaptation and reproductive isolation in natural populations, we conducted a novel reciprocal transplant experiment involving outbred lines, where alternative arrangements of the inversion were reciprocally introgressed into the genetic backgrounds of each ecotype. Our results demonstrate for the first time in nature the contribution of an inversion to adaptation, an annual/perennial life-history shift, and multiple reproductive isolating barriers. These results are consistent with the local adaptation mechanism being responsible for the distribution of the two inversion arrangements across the geographic range of *M. guttatus* and that locally adaptive inversion effects contribute directly to reproductive isolation. Such a mechanism may be partially responsible for the observation that closely related species often differ by multiple chromosomal rearrangements.

## Abstract 2

A set of experiments demonstrates the involvement of a chromosomal inversion in the adaptive transition between annual and perennial ecotypes of the yellow monkeyflower, *Mimulus guttatus*

## Author Summary

Genome rearrangements that change the order of genes along a chromosome are known as inversions and have long been hypothesized to be involved in the origin of species. Yet the way inversions contribute to adaptation and speciation remains mysterious. In this study, we identified a geographically widespread adaptive inversion polymorphism in the yellow monkeyflower, *Mimulus guttatus*. One arrangement of the inverted region is found in an annual ecotype of this species that lives in Mediterranean habitats characterized by reduced soil water availability in the summer. The other arrangement appears in a perennial ecotype that lives in habitats with high year-round soil moisture. The inversion was observed to influence morphological and flowering time differences between these ecotypes across most of western North America. To test whether the inversion polymorphism contributes to adaptation and reproductive isolation, we conducted a field experiment by breeding plants to reciprocally swap the alternative chromosomal arrangements between the annual and perennial genetic backgrounds. We demonstrated that this inversion polymorphism contributes to local adaptation, the annual/perennial life-history transition, and three reproductive isolating barriers. These results are consistent with the theory that adaptation to local environments can drive the spread of chromosomal inversions and promote speciation.

## Introduction

Closely related species frequently differ by chromosomal rearrangements such as inversions [1],[2], and these chromosome differences have long been thought to play a critical role in adaptation and speciation [3]–[14]. Inversions can directly cause hybrid sterility in chromosomal heterozygotes through the production of unbalanced gametes due to crossing over in the rearranged regions during meiosis [1],[5],[7],[10]. A more recent view is that the main evolutionary importance of inversions is that they suppress recombination between alternative chromosomal arrangements in hybridizing populations, and as a result become associated with genes involved in local adaptation or reproductive isolation [8],[12],[15],[16]. There are several distinct ideas of how such associations may arise. First, if initially allopatric, incompletely isolated populations secondarily come into contact and begin to hybridize, then reproductive incompatibility alleles from each population will be purged by natural selection unless prevented by recombination suppression in inverted regions [8],[17],[18]. Second, differential purging of incompatibility alleles in co-linear regions versus maintenance of incompatibilities within inversions might also generate selection for prezygotic isolating alleles to accumulate through the process of reinforcement, especially if they are linked to the inversions [8],[19]–[21]. Third, alleles that are adaptive within one species' genetic background, but that cause hybrid lethality or sterility when introgressed into another species, can continue to accumulate in inversions that differentiate the species, despite migration and hybridization [9].

However, these theories, which emphasize the resistance of inversions to homogenization by hybridization, or the differential accumulation in inversions of hybrid incompatibility factors, cannot readily explain how inversions initially establish or why they often appear linked to loci involved in climatic or habitat adaptation (reviewed in [16]). As long as selection is stronger than migration between habitats, alleles involved in local adaptation will be maintained at locally high frequencies regardless of local recombination rates [22],[23]. Alleles at such loci can actually cause inversions to invade locally adapted populations when they “capture” linked alleles that are already near fixation as a result of migration-selection balance [12]. In this scenario, an inversion that happens to include locally adaptive alleles at two or more loci will rapidly spread through populations where those alleles are favored, because it has a fitness advantage over all recombining haplotypes [12]. Thus, provided at least some migration, inversions that capture multiple locally adapted alleles are predicted to differentiate diverging populations. In contrast to older ideas that explained the maintenance of polymorphic inversions via coadapted gene complexes [4],[24],[25], the local adaptation mechanism does not depend on epistasis between the locally adaptive alleles. If inversions that differentiate hybridizing populations or species tend to be associated with multiple loci involved in local adaptation, then they should also contribute to prezygotic isolation (i.e., immigrant inviability) and/or extrinsic postzygotic isolation.

Numerous studies have found associations between putatively adaptive phenotypes and inversions (reviewed in [16]). Many others have found predictable distributions of inversion polymorphism along environmental clines [12],[24],[26]–[30] and predictable shifts in the frequency of inversions over the course of a season [31]–[33]. One of the best examples is the *In(3R)Payne* inversion in *Drosophilia melanogaster* that is latitudinally distributed along clines around the world and has recently shifted its distribution in response to global climate change [28]. However, definitive demonstration of the involvement of an inversion in adaptation and ecological reproductive isolation requires the following: (1) Inversion polymorphism must be shown to be partitioned between reproductively isolated groups, (2) replicable links must be made between an inversion and the phenotypes responsible for isolation, and (3) field experiments must be conducted to show that the inversion contributes to adaptation and reproductive isolation in nature. While previous studies have found associations between inversions and traits involved in ecological reproductive isolation [27],[34],[35], to our knowledge there are no reports of field experiments that directly determine the relative contribution of an inversion to local adaptation or whether putative adaptive inversions actually cause ecological reproductive isolation in natural populations.

The yellow monkeyflower *Mimulus guttatus* is an excellent genomic model system [36] to test whether inversions are involved in habitat-mediated adaptation and ecological reproductive isolation. Widespread inland annual and coastal perennial ecotypes of *M. guttatus* have been shown to be locally adapted to their contrasting habitats and, as a result, reproductively isolated due to strong ecological prezygotic reproductive isolating barriers quantified in the field [37]–[39]. An adaptive flowering time shift underlies a large portion of the local adaptation and reproductive isolation in this system through both temporal isolation and selection against immigrants between habitats [38]. Selection against immigrants is particularly strong in inland annual habitat where transplanted late-flowering coastal perennial plants fail to flower before the onset of the hot seasonal summer drought (Figure S1) [37],[38],[40]. In contrast, early-flowering inland annual plants are at a disadvantage in coastal habitat as they invest more resources in reproduction instead of growth and thus fail to take advantage of year-round soil moisture and cool foggy conditions [41] responsible for the native coastal perennial life-history [37]. This life-history shift involving growth and reproduction is controlled by a complex genetic architecture including a few large-effect quantitative trait loci (QTL) (20%–30% of ecotypic trait divergence) [42]. More recent mapping using greater numbers of co-dominant markers has revealed that one of those large-effect loci appears to be located in a region of linkage group eight with unusually large numbers of completely linked markers, indicating the potential involvement of a chromosomal rearrangement [39],[40].

Here, we evaluate the relative contribution of the chromosomal inversion on linkage group eight to habitat adaptation and consequent ecological reproductive isolation between geographically widespread perennial and annual ecotypes of *M. guttatus*. First, we establish that the suppressed recombination on linkage group eight in our mapping population is caused by an inversion polymorphism, with reversed orders of genetic markers in the annual and perennial parents. Furthermore, we show that this inversion polymorphism is geographically widespread and appears to be perfectly associated with the divergent life-histories, suggesting the involvement of a chromosomal inversion in differentiation of the ecotypes. Next, we confirm that the inversion has consistent effects on flowering time divergences in multiple independent population crosses through replicated QTL analysis. Finally, we quantify the effects of the inversion on phenotypes and local fitness under natural field conditions through the incorporation of outbred introgression lines into a reciprocal transplant experiment. This design allowed us to demonstrate that the effects of the inversion polymorphism are robust to genetic background and contribute both to adaptation and to ecological reproductive isolation across habitats.

## Results

### Inversion Polymorphism Associated with Inland Annual and Coastal Perennial Habitat

**Figure 1:**
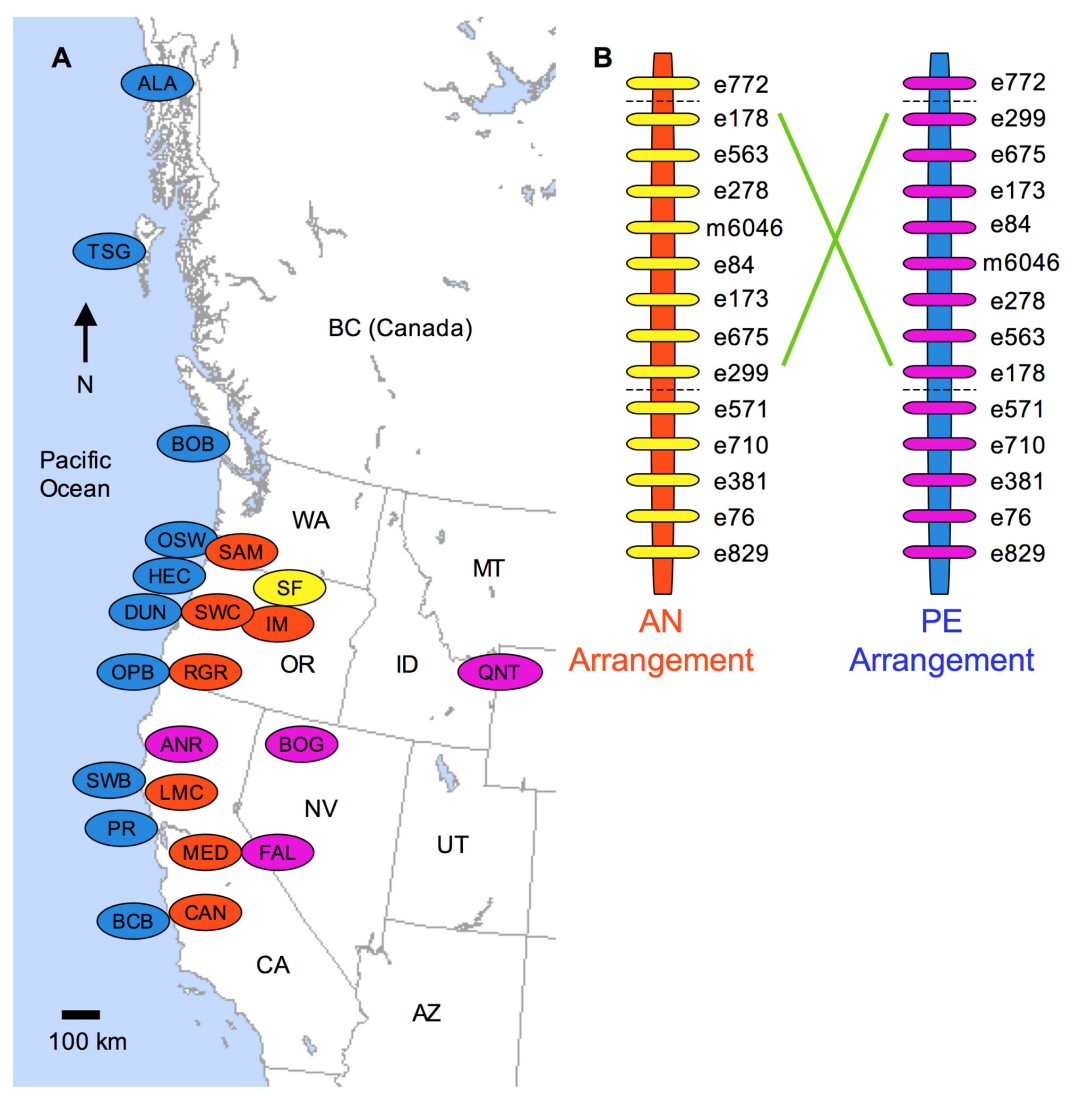
Geographic distribution of the chromosomal inversion. (A) Map of western North America with the locations of populations of coastal perennials (blue), inland annuals (orange), and inland perennials (purple), as well as obligate self-fertilizing species *M. nasutus* (yellow). (B) Marker order of the AN and PE inversion arrangements along linkage group eight. Inland annuals and *M. nasutus* had the AN arrangement while coastal and inland perennials all had the PE arrangement.

Previously, we observed substantially suppressed recombination among markers tightly linked to a large-effect, pleiotropic QTL on linkage group eight [42] in a recombinant inbred line (RIL) mapping population derived from the IM (inland annual) and DUN (coastal perennial) populations of central Oregon [39],[40]. This suppression of recombination has not been observed in crosses between annual parents [42]. To determine if the suppression of recombination found in the IM×DUN cross is due to an inversion, we constructed multiple F2 mapping populations by crossing within and among populations of the annual and perennial ecotypes (Figure 1; Tables S1, S2). If alternative chromosomal arrangements are fixed within the inland annual and coastal perennial ecotypes, then there should be suppressed recombination in all inter-ecotype crosses, whereas there should be much larger map distances in crosses within each ecotype. Critically, marker order should be reversed in crosses among perennial populations as compared to crosses among annual populations. We did not observe any recombinants between the consistently polymorphic markers e299 and e278 (Figure 1B) in the 429 inland annual×coastal perennial F2s screened (48–96 per population pair; Table S2). Ongoing mapping experiments involving crosses within the annual IM population consistently exhibit map distances between those markers ranging from 23.3 to 32.0 cM (linkage maps available at mimulusevolution.org). Genotyping of additional markers in the annual×perennial crosses (e173, e178, and/or e675 depending on which were polymorphic) in the region confirmed the lack of crossing over observed in the presumed inverted region. Since no cross over products were observed in more than 2,270 effective meioses of our study (801 F2s and 334 RILs; Table S2), the 95% confidence interval for the recombination frequency of the inversion includes a maximum 0.15%.

In contrast, substantial recombination was observed among these markers in four crosses (Table S2) among six coastal perennial populations from California (SWB), Oregon (HEC and DUN), Vancouver Island, BC (BOB), the Queen Charlotte Islands, BC (TSG), and southeastern Alaska (ALA). The resulting genetic maps also confirmed that marker order is reversed in that region of linkage group eight in the inland annual populations relative to coastal perennial populations. For purposes of clarity, we denote the inland annual arrangement as AN and the coastal perennial arrangement as PE (Figure 1).

We observed recombination in the inverted region in the cross between the annual IM population and two other inland annual populations, LMC and MED (Table S2). The marker order for these crosses was the same as that previously observed in crosses within the IM population. An annual obligate self-fertilizing species *M. nasutus* (SF population), thought to be derived from *M. guttatus* [43], was also found to have the AN arrangement (Figure 1B, Table S2).

Markers in the inverted region, which are completely linked in crosses between ecotypes, span a genetic map region between the most distant markers e178 and e299 of at least 33 cM in previous [39] and ongoing mapping studies within the annual ecotype. This is about 2% of the estimated total genetic map length of 1,500–2,000 cM (markers and linkage maps are available at www.mimulusevolution.org). Markers from within the inversion are located on two genome sequence scaffolds (11 and 233) of the recently released draft *M. guttatus* genome assembly v1.0 (sequence data available at www.phytozome.net). While the inversion breakpoints are as yet unknown, the inversion encompasses at least 2.22 Mbp, including at 68.6% of scaffold 11 (2.98 Mbp) and 48.8% of scaffold 233 (0.37 Mbp), or less than 1% of the 450 Mbp estimated genome size of *Mimulus*. This region appears to contain 362 genes identified by the current v1.0 genome annotation.

### Inland and Coastal Perennials Share the Same Inversion Arrangement

Perennial life-history is not limited to coastal perennial populations of *M. guttatus*. While many inland populations of *M. guttatus* are annual, numerous inland perennial populations are found in areas of year-round soil moisture, such as on the edge of lakes or in rivers, hot springs, and alpine habitats [44],[45]. Coastal and inland perennial *M. guttatus* populations have many traits in common [44],[45], but the relationship of these ecotypes is yet to be evaluated.

To determine if inland perennial populations have the same chromosomal inversion arrangement (PE) as coastal perennial populations, we conducted independent crosses between the DUN coastal perennial populations and four inland perennial populations, from as far as 1,000 km from the Pacific Ocean (Figure 1A). Patterns of recombination suppression and marker order indicate that all four of these inland perennial populations have the PE arrangement (Figure 1B, Table S2).

### Phenotypic Effects of the Inversion Polymorphism Are Replicable Over a Wide Geographic Range

**Figure 2:**
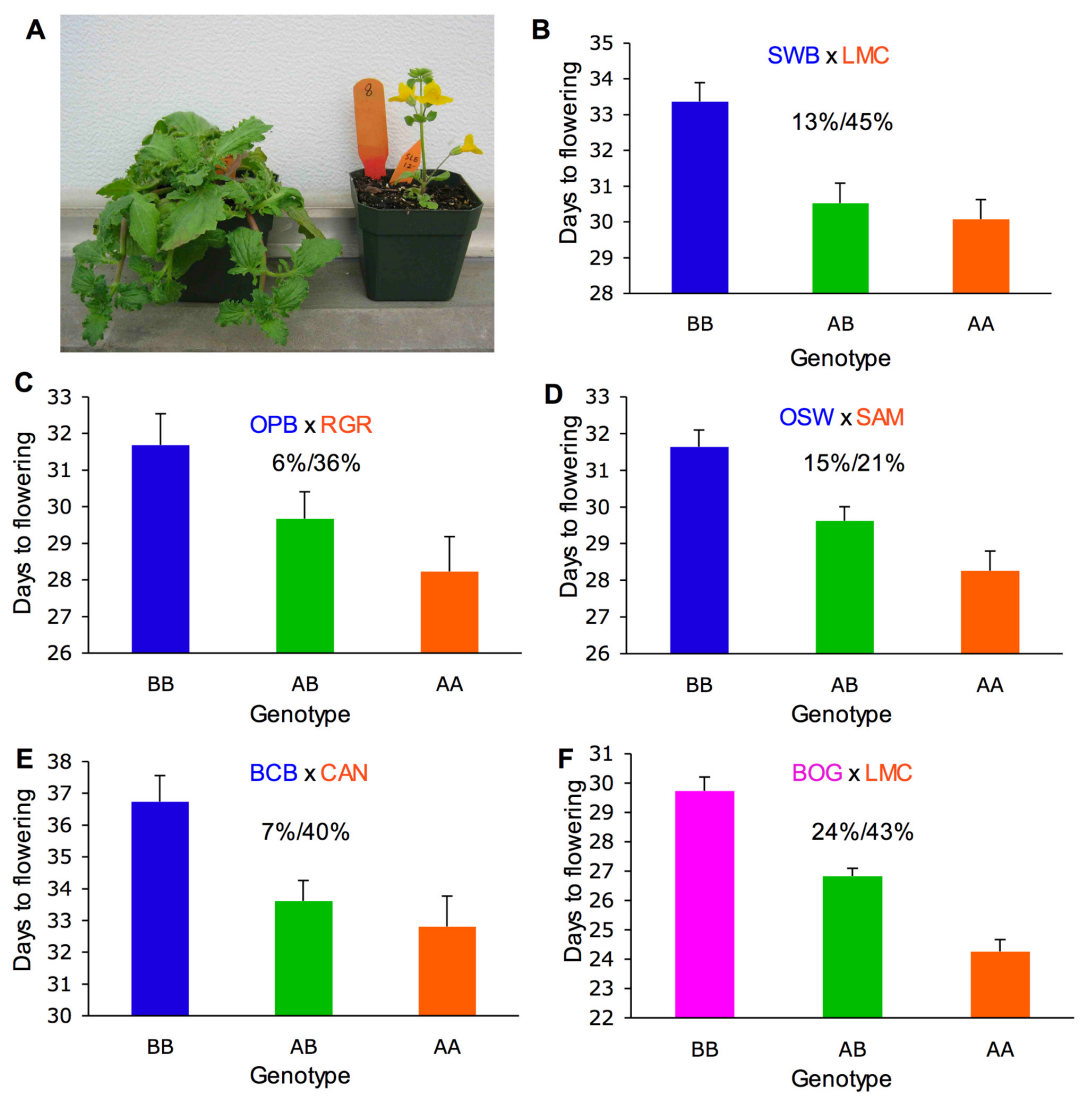
Replicated effect of the inversion locus. (A) F2 progeny with parental ecotypic phenotypes, from a cross between the SWB (coastal perennial) and LMC (inland annual) populations. (B–E) Effect of the inversion on flowering time in four independently derived F2 mapping populations created through crosses between independent inland annual and coastal perennial populations. (F) Effects of the inversion on flowering time in cross between inland annual and inland perennial populations. The mean flowering times (±1 SE) of F2s that were homozygous for the AN arrangement (AA), heterozygous (AB), and homozygous for the PE arrangement (BB) at Micro6046 are indicated. The percentage of F2 variance/parental divergence explained by the inversion is presented above each bar graph. Note: *y*-axes do not originate at zero.

To determine if the inversion contributes to the divergence of morphology and flowering time over the range of the annual and perennial ecotypes, we conducted replicated QTL mapping experiments using an outbred breeding design. Progeny resembling annual and perennial parental types were observed in all four inland annual×coastal perennial mapping populations in the F2 generation (Figure 2A). However, the degree to which parental phenotypes were recovered varied among traits and crosses (Figures S2, S3; Table S3).

The inversion consistently affected the composite of morphological traits (MANOVA) in the CAN×BCB (Wilks' λ = 0.701, *F_8,322_* = 7.808, *p*<0.0001), LMC×SWB (Wilks' λ = 0.811, *F_8,292_* = 4.01, *p* = 0.0002), RGR×OPB (Wilks' λ = 0.770, *F_8,234_* = 4.074, *p* = 0.0001), and SAM×OSW (Wilks' λ = 0.804, *F_8,288_* = 4.153, *p* = 0.0001) mapping populations. The inversion also explained a large percentage of the parental divergence (21%–45%) and F2 variance (*R^2^* = 0.06–0.15) in flowering time in all four of these mapping populations (Figure 2, Table 1).

**Table 1:** The effects of the inversion locus on flowering time and morphological traits in the greenhouse.

	Flowering Time			Stem Thickness			Internode Length			Corolla Length			Corrola Width			Aboveground Roots		
Cross	2a	d	2a/diff	2a	d	2a/diff	2a	d	2a/diff	2a	d	2a/diff	2a	d	2a/diff	2a	d	2a/diff
CAN×BCB (*N* = 167)	3.93**	−1.16	0.40	0.70*	0.04	0.13	−5.60	6.22	−0.23	5.61****	0.36	0.32	2.74****	0.16	0.26	0.73****	−0.14	0.32
LMC×SWB (*N* = 153)	3.29****	−1.20	0.45	0.55*	−0.09	0.18	−19.39***	5.98	−0.33	2.04***	−0.47	0.20	0.81*	−0.49	0.10	N/A	N/A	N/A
RGR×OPB (*N* = 123)	3.47*	−0.28	0.36	0.65**	0.21	0.27	2.76	1.15	0.10	4.31****	0.59	0.49	2.65****	0.235	0.30	1.12**	−0.26	0.21
SAM×OSW (*N* = 151)	3.43****	−0.36	0.21	0.65**	−0.07	0.18	2.96	0.49	0.12	2.62**	−0.43	0.56	1.43**	−0.23	0.23	N/A	N/A	N/A

For each trait: the additive effect (2a), dominance deviation (d), and the proportion of the parental population divergence explained (2a/diff).

*p<0.05, **p<0.01, ***p<0.001, ****p<0.0001.

Given that both the AN and PE arrangements of the inversion are found in inland regions, we hypothesized that the inversion would also have an effect on flowering time divergence between inland annual and inland perennial populations. To test this, we scored flowering time in a F2 population created through a cross between lines from the inland annual LMC and inland perennial BOG populations. The inversion significantly explained 43% of the parental divergence and nearly a quarter (*R^2^* = 0.24) of the F2 variance in flowering time in this inland annual×perennial cross (Figure 2F; *F*_2,266_ = 42.02; *p*<0.0001).

### Inversion Polymorphism Contributes to Trait Divergence, Life-History Divergence, and Fitness in the Field

**Figure 3:**
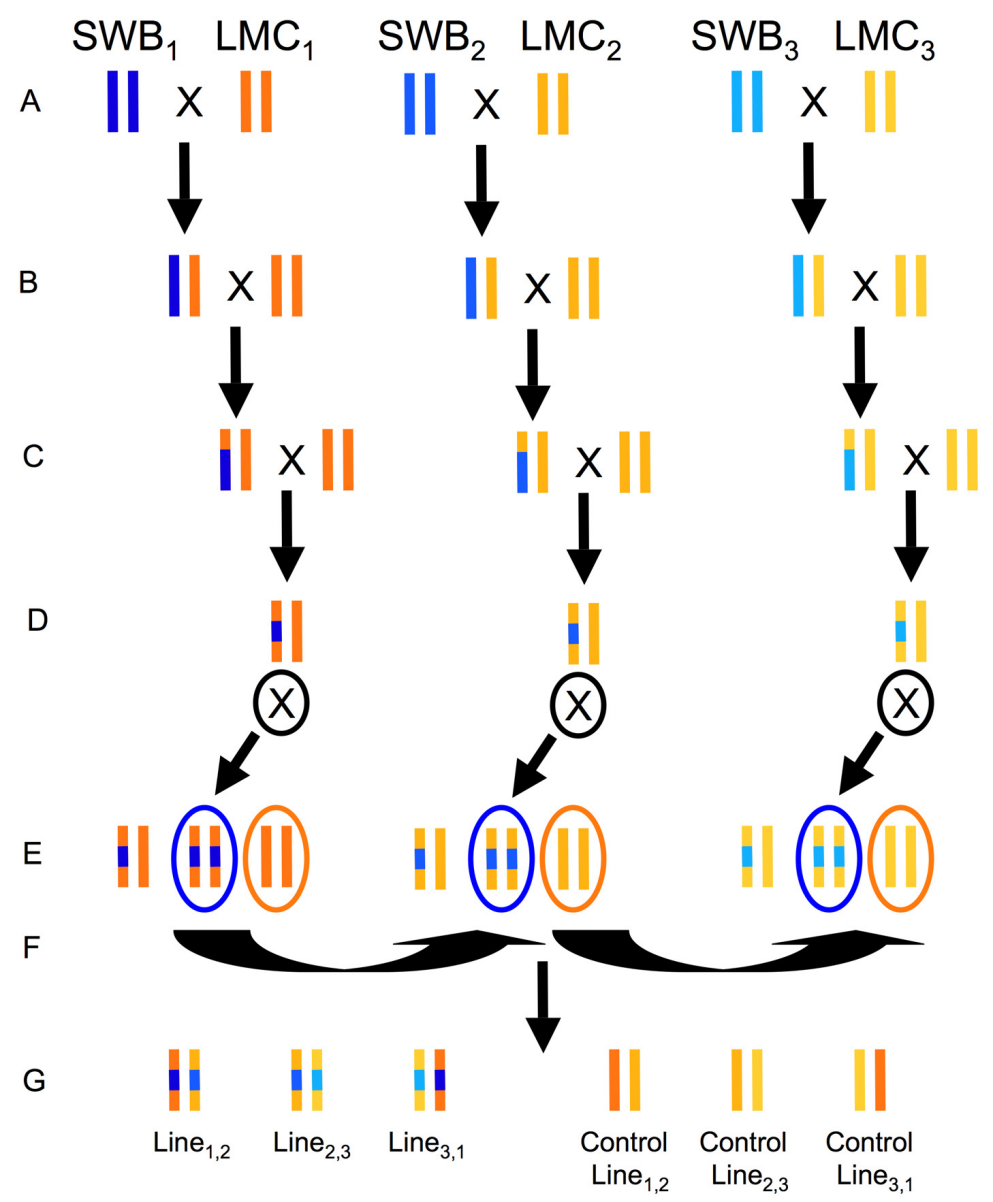
Breeding design for creation of backcross introgression lines. Crossing design for production of backcross lines with the LMC (shades of yellow/orange) genetic background. Breeding of plants with SWB (shades of blue) genetic background not shown. Note that the size of introgressed region around the inversion should vary among lines due to different recombination locations during breeding. Different shades are used to indicate that original parental inbred lines have a unique genetic composition. (A) Three pairs of independently derived inbred LMC and SWB lines were crossed to create F1 progeny. (B) F1s backcrossed to parental inbred lines. (C) Marker-assisted selection used for four generations of backcrossing to move the inversion into alternate genetic backgrounds. (D) Heterozygous lines were self-fertilized. (E) Backcross lines that are homozygous with (blue oval) and without (orange oval) the introgressed inversion arrangements were selected for further breeding. (F) Intercrosses conducted among the three independent groups to create outbred backcross lines with and without the introgressed inverted region. (G) Backcrossed lines now ready to be planted into field reciprocal transplant experiment.

To determine the relative contribution of the inversion polymorphism to local adaptation and ecological reproductive isolation in the field, we conducted a reciprocal transplant experiment that included an outbred set of backcross and parental lines (Figure 3). This experiment was designed to allow us to compare, in the field, the performance of alternative inversion homozygotes in each of the two ecotypes' genetic backgrounds to each other and to the original parental ecotypes. In order to ensure realistic fitness comparisons without the potentially confounding effects of inbreeding depression, we used a novel crossing design that ensured that all experimental plants were outbred despite having particular combinations of genetic background and inversion genotype.

To construct the outbred set of backcross lines, the AN and PE arrangements of the inversion were introgressed, by repeated backcrossing, into the genetic and cytoplasmic backgrounds of the alternative ecotype. Importantly, we initiated breeding with three independent pairs of coastal perennial and inland annual inbred lines derived from the SWB and LMC populations of northern California. F1 progeny of these three interpopulational crosses were backcrossed to each of their respective LMC and SWB parental inbred lines, for a total of six backcross populations (two reciprocal backcrosses for each of the three original interpopulational crosses). In each backcross population, markers in the inverted region were genotyped to identify a single inversion heterozygote to be used in an additional generation of backcrossing to the recurrent parental inbred line. This procedure was continued for a total of four backcross generations.

After the fourth backcross generation, a single inversion heterozygote in each of the six backcross populations was self-fertilized and the resulting progeny were genotyped to identify a single homozygote of each arrangement. For each of the two genetic backgrounds, we then intercrossed the three AN homozygotes to each other, and the three PE homozygotes to each other, to generate plants that had one of the four combinations of genetic background and homozygosity for a particular chromosomal arrangement but were outbred throughout the genome. To generate outbred parental lines, the three annual inbred parental lines were intercrossed to each other, as were the three perennial inbred parental inbred lines (Figure 3). Finally, both outbred parental lines and the four outbred backcross lines were planted at two field sites located in inland annual (Figure S4) and coastal perennial habitat (Figure S5).

As in previous studies [37],[38], there was a highly significant genotype×environment interaction (*p*<0.0001; Tables 2, S4) across habitats for inland annual and coastal perennial parents based on the composite of two fitness traits: survival to flowering and number of flowers produced per plant. Local adaptation was very strong, with native parental types producing 60 times more flowers in inland annual habitat and 13 times more flowers in coastal perennial habitat (Table 2). Analysis of the introgression lines also revealed a highly significant effect of genetic background and genetic background×site interaction (both *p*<0.0001; Tables 3, S4). In terms of the inversion, there were significant arrangement×site and arrangement×genetic background interaction effects on fitness (both *p*<0.0001; Table 3). However, the three-way interaction of arrangement×site×genetic background was not significant (*p*>0.05).

**Table 2:** Effects of the inversion locus on components of fitness in the field reciprocal transplant experiment.

Field Site	Inversion Orientation: Genetic Background	*N* a	Days to Flower^b^	Survival to Flower^c^	Flowers Produced^d^	Expected Flowers^e^	End of Season^f^	Yet to Flower^g^
Boonville (Inland Annual)	Inland parent	204	52.04 (0.61)	89.71	14.78 (0.88)	13.27 (0.42)	0.00	NA
	PE arrangement: Annual Genetic Background	178	57.60 (0.69)	87.08	9.45 (0.65)	8.24 (0.35)	0.00	NA
	AN arrangement: Annual Genetic Background	191	53.59 (0.61)	94.76	11.70 (0.64)	11.11 (0.28)	0.00	NA
	Coastal parent	199	77.57 (1.35)	6.03	3.00 (0.72)	0.22 (0.11)	0.00	NA
	AN arrangement: Perennial Genetic Background	195	73.49 (0.92)	51.28	5.26 (0.48)	2.82 (0.29)	0.00	NA
	PE arrangement: Perennial Genetic Background	201	82.54 (3.00)	6.47	3.77 (0.57)	0.29 (0.08)	0.00	NA
Manchester (Coast Perennial)	Inland parent	195	80.56 (2.45)	9.23	4.44 (0.85)	0.45 (0.16)	0.00	NA
	PE arrangement: Annual Genetic Background	184	90.22 (2.88)	12.50	6.43 (1.53)	0.85 (0.17)	0.00	NA
	AN arrangement: Annual Genetic Background	190	86.00 (3.19)	8.95	4.53 (1.17)	0.39 (0.10)	0.00	NA
	Coastal parent	191	138.08 (2.91)	35.07	16.82 (6.02)	5.80 (0.50)	38.22	43.83
	AN arrangement: Perennial Genetic Background	191	118.14 (2.50)	46.32	12.00 (2.33)	5.46 (0.36)	10.53	5.00
	PE arrangement: Perennial Genetic Background	195	139.46 (3.69)	34.87	12.12 (1.92)	4.06 (0.33)	36.92	45.21

a Number of individuals planted per genotype.

b Mean (±SE) number of days to first flower.

c Percentage of plants surviving to flower.

d Mean (±SE) number of flowers produced per plant surviving to flower.

e Expected number of flowers (±SE) per plant at start of experiment calculated with ASTER.

f Percentage of plants still alive at the end of the first field season.

g Percentage of plants surviving to the end of the first season that had not yet flowered.

**Table 3:** Analysis of effect of genetic background, field site, and inversion locus by ASTER.

Factor Tested	Null *df*	Alternative *df*	Null Deviance	Alternative Deviance	Test *df*	Test Deviance	Test *p* Value
Genetic background	3	4	−13,975.0	−14,035.8	1	60.8	<0.0001
Genetic background×site	4	5	−14,035.8	−14,658.9	1	623.0	<0.0001
Inversion arrangement	5	6	−14,658.9	−14,722.5	1	63.7	<0.0001
Inversion arrangement×site	6	7	−14,722.5	−14,755.4	1	32.9	<0.0001
Inversion arrangement×genetic background	7	8	−14,755.4	−14,775.0	1	19.6	<0.0001

Factors tested with ASTER using the composite of two dependent components of fitness, survival to flowering, and number of flowers produced, with the following directional graph: 1 -> survival to flowering -> number of flowers produced. All factors are tested by likelihood ratio tests using nested null models.

**Figure 4:**
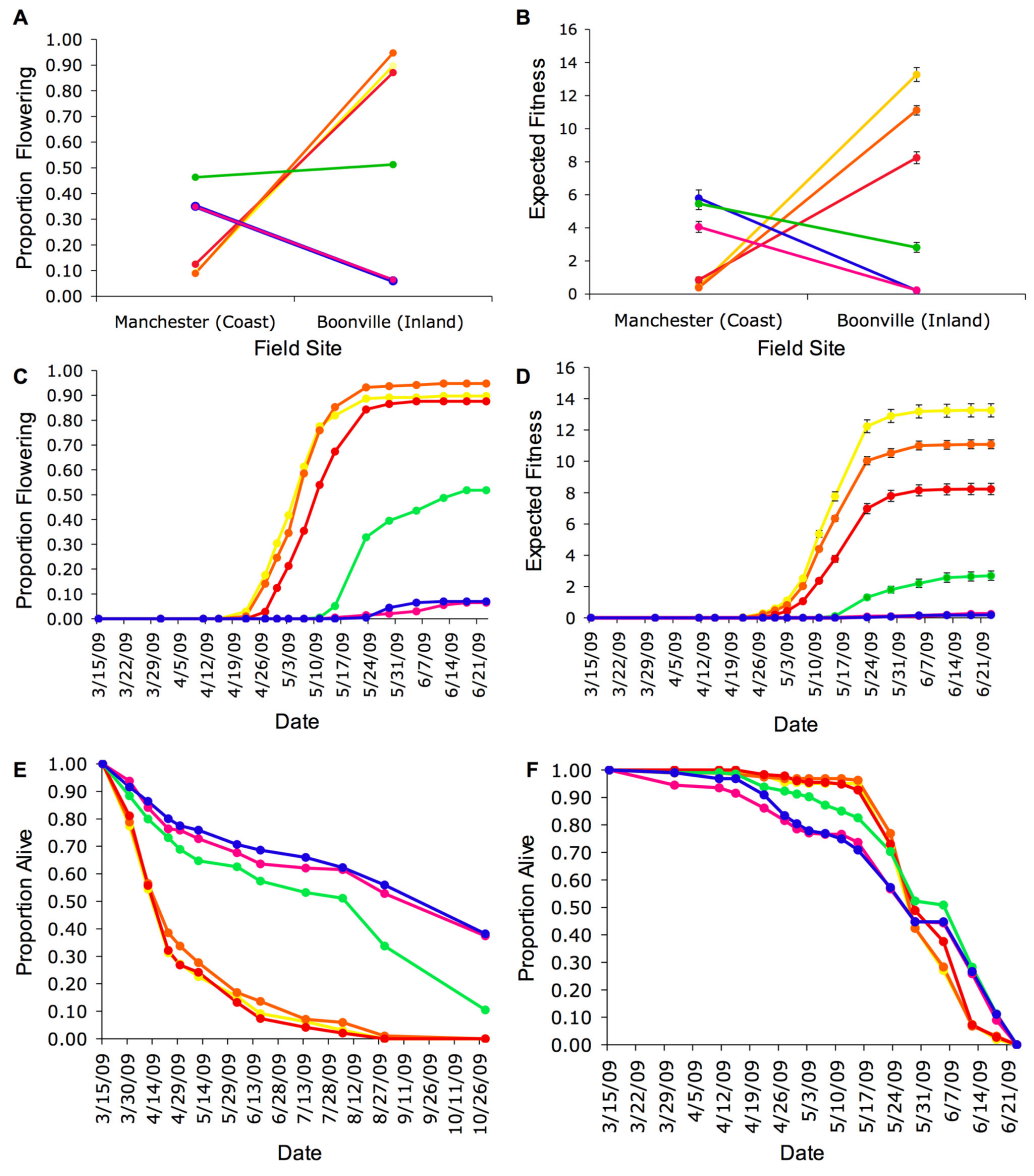
Effects of the inversion locus across field sites in different genetic backgrounds. (A) Proportion of plants surviving to flower and (B) expected fitness produced per plants across field sites. Values plotted are maximum likelihood estimates ±1 SE calculated with ASTER. (C) Cumulative proportion of plants surviving to flower and (D) expected fitness per individual at the inland field site. Survival over time at the (E) coastal perennial and (F) inland annual field sites. Parental lines: yellow, inland annual parent; blue, coastal perennial parent. Backcross lines: orange, PE arrangement in annual genetic background; red, AN arrangement in annual genetic background; green, AN arrangement in perennial genetic background; pink, PE arrangement in perennial genetic background.

At the inland field site, the inversion explained similar amounts of the divergence in parental flower production in the inland (21.99%) and coastal (19.39%) genetic backgrounds (Figure 4B). The effects of the inversion on flower production at the inland field site can be attributed largely to its effect on flowering time. Across the backcross lines, plants with the PE arrangement initiated flowering 6.54 d later than plants with the AN arrangement (*F_1,445_* = 23.10; *p*<0.0001). Later flowering plants produced fewer flowers before the summer drought made further survival impossible (Figure 4). This effect of flowering time and fitness was most dramatic in the coastal genetic background where survival to flowering was eight times greater for plants with the AN versus PE arrangement (Figure 4; Table 2).

At the coastal field site, the inversion had a significant 12.77 d effect on flowering time among the backcross lines (*F_1,192_* = 8.34; *p* = 0.0043), where plants with the AN arrangement flowered earlier (Figure 4, Table 2). However, in contrast to the inland site, earlier flowering only translated into slightly greater expected fitness for the AN arrangement versus the PE arrangement in the coastal perennial genetic background (Figure 4, Table 2). Individuals with the PE arrangement produced 2.13 times as many flowers as those with the AN arrangement in the foreign inland annual genetic background (Table 2).

The inversion had significant effects on patterns of survival over the course of the season at both the coastal (*p* = 0.0044) and inland (*p* = 0.0124) field sites (Table 4; Figure 4). At the coastal site, plants with the PE arrangement had a 3.5 times greater survivorship (69% of the parental divergence) to the end of the first season (e.g. first rain of the 2009/2010 wet season) than plants with the AN arrangement (Figure 4F). Nearly half of the plants that survived to the end of the first season, and were homozygous for the PE arrangement, did not flower during the 2009 field season (coastal perennial parent = 45% and PE arrangement in perennial genetic background = 44% versus AN arrangement in perennial genetic background = 5%). Thus, plants with the PE arrangement allocated all of their resources to growth instead of reproduction in the first season at nearly 10 times the rate of those with the AN arrangement.

**Table 4:** Effects of inversion on survival over the field season at each field site.

Survival by Field Site	Null *df*	Alternative *df*	Null Deviance	Alternative Deviance	Test *df*	Test Deviance	Test *p* Value
Inland annual site	16	17	3,270.6	3,264.3	1	6.3	0.0124
Coast perennial site	12	13	3,265.8	3,257.7	1	8.1	0.0044

Tests of the inversion locus effect on survival over the course of the season using ASTER, where survival is modeled with the following directional graph: 1 -> survival at census one -> survival at census two -> … -> survival at census x, where x = 16 for the inland field site and 11 for the coastal field site.

### Contribution of Inversion Polymorphism to Reproductive Isolating Barriers

To quantify the contribution of the inversions to ecological reproductive isolation, we modified methods that we developed previously [38],[46]. Here, reproductive isolation ranges from one (complete isolation) to zero or even negative (no reproductive isolation).

As in a previous study [38], reproductive isolation due to differences in flowering time (temporal isolation) between habitats (*RI_TBH_*) was near complete, with very little overlap in flowering time between the ecotypes across habitats (Table 5). The inversion polymorphism's effect on flowering time was not great enough to overcome the large differences in flowering between the coastal perennial and inland annual genetic backgrounds across habitats. Thus, the inversion did not contribute much to between habitat isolation caused by flowering time differences (Table 5).

**Table 5:** Overall strength of reproductive isolating barriers and individual contribution by the inversion.

	Between Ecological Races^a^		Inversion Contribution^b^	
Reproductive Isolating Barrier	Coast	Inland	Coast	Inland
Temporal flowering isolation between habitats (*RI_TBH_*)	0.999	0.997	0.001	−0.008
Selection against immigrants (*RI_I_*)	0.922	0.983	0.079	0.150
Temporal flowering isolation in sympatry (*RI_TS_*)	0.810	0.166	0.213	0.026
Extrinsic postzygotic isolation (*RI_EP_*)^c^	−1.801/0.538	0.233	−0.241/0.690	0.216

a Overall reproductive isolation between inland annual and coastal perennial ecological races at coast or inland field sites.

b The individual locus contribution to reproductive isolation by the inversion at the coast or inland field sites.

c Quantified separately at the coast field site for two fitness components: expected number of flowers/survival to second season.

Ecological reproductive isolation due to selection against immigrants (*RI_I_*) was very strong in both inland and coastal habitats (Table 5). Comparisons of the fitness of the AN and PE arrangements in the foreign coastal genetic background, in inland habitat, revealed that the inversion had a moderate individual locus effect on selection against immigrants (*RI_I,Inversion_* = 0.150). The inversion had less effect on the difference in fitness between inversion arrangements for migrants of the inland annual genetic background to coastal habitat, for an individual locus contribution of *RI_I,Inversion_* = 0.079.

Differences in flowering time can also prevent gene flow into native populations from foreign pollen donors that successfully survive to flower in their non-native habitat. Such temporal flowering isolation in sympatry (*RI_TS_*) was high in coastal perennial habitat but low in inland annual habitat (Table 5), where the summer drought severely truncated the flowering distribution of the foreign coastal plants. The inversion's effect on a shift in the flowering time distribution between arrangements in the inland annual genetic background resulted in a considerable contribution (*RI_TS,Inversion_* = 0.213) to temporal reproductive isolation in coastal perennial habitat. The inversion had little effect (*RI_TS,Inversion_* = 0.026) on sympatric temporal isolating barrier in inland annual habitat (Table 5).

Finally, we quantified the effect of the inversion on extrinsic postzygotic isolation (*RI_EP_*) through the comparisons of flower production between arrangements of the genetic background native to that habitat. Previously [38], we found F1 generation extrinsic postzygotic isolation based on first season flower production to be weak in inland annual habitat but ranged widely in coastal perennial habitat, depending on the component of fitness measured (Table 5). After the F1 generation, extrinsic postzygotic isolation is an individual locus phenomenon because of recombination. Thus, extrinsic postzygotic isolation of advance generation hybrids is defined here as the tendency of inversion polymorphism to remain restricted between two ecotypes after hybridization, as a consequence of external selection. Extrinsic postzygotic isolation of the inversion locus, based on first season flower production, was moderate in inland annual habitat (*RI_EP,Inversion_* = 0.216) but negative in coastal habitat (RI = −0.241). However, extrinsic postzygotic isolation of the inversion based on multiseason survival in coastal perennial habitat was strong (*RI_EP,Inversion_* = 0.690).

## Discussion

Overall, our study found that a chromosomal inversion polymorphism contributes to adaptive divergence and reproductive isolation between annual and perennial ecotypes of *M. guttatus*. The AN arrangement of the inversion was consistently found in annual populations and the PE arrangement found in perennial populations distributed over a wide swath of western North America. The inversion polymorphism affected traits associated with this life-history transition across replicated crosses and the genetic backgrounds of both ecotypes, while contributing to local adaptation, perenniality, and three ecological reproductive isolating barriers under natural field conditions.

### Inversions and Adaptation

Inversions are frequently distributed geographically along environmental clines [24],[27]–[30],[47],[48] or exhibit predictable seasonal changes in allele frequency [31]–[33], while putatively adaptive traits such as phenological shifts, desiccation tolerance, and thermal tolerance often map to inversions (reviewed in [16]). For example, inversions are known to contribute to the divergence in timing of overwintering pupal diapause between host races of Tephritid fruit flies, *Rhagoletis pomonella* [34],[35]. In *M. guttatus*, the geographic distribution of the AN and PE arrangements appears to be dictated by the availability of soil moisture in summer months across western North America (Figure S1) [37],[38]. However, the distribution of inversion polymorphism is not clinal as in many other organisms. Rather the distribution appears to be an overlapping mosaic of discrete annual and perennial populations that contain the AN or PE arrangements depending on local environmental conditions. Further geographic sampling will be necessary to establish the full range of the two inversion arrangements.

The inversion investigated here is involved in a classic life-history shift in plants that is an adaptive response to differences in the seasonal availability of water resources [49]–[53]. The AN arrangement of the inversion promotes rapid flowering over sustained vegetative growth, leading to an annual life-history strategy that allows for avoidance of summer drought. In contrast, the PE arrangement of the inversion promotes greater vegetative growth early in the season, followed by summer flowering and survival into subsequent years, and therefore a perennial life-history.

### Selective Mechanisms Underlying the Geographic Distribution of the Inversion

Alternative inversion arrangements are expected to invade ecologically divergent habitats if they capture two or more loci with locally adapted alleles that are already near fixation, despite gene flow, as a result of local selective pressures [12]. Our geographic data suggest that alternative arrangements of the inversion are restricted to annual versus perennial habitats. The finding that inland perennial populations as well as coastal perennial populations have the PE arrangement of the inversion and that the early flowering selfing species *M. nasutus* has the AN arrangement further suggests that the distribution of the inversion is a function of the availability of soil moisture during summer months.

Year-round water availability, typical of the perennial habitats, allows plants that survive to the second season to become well-established [37],[40]. These established plants may have an advantage in wetter habitats, such as the northern Pacific coast of North America, because they are primarily composed of other perennial plant species that may shade out and compete below ground except in areas of natural disturbance. Natural landslides and deer trampling in the coastal perennial habitat continually creates disturbed habitat for new seedlings recruitment [54]. Our field study mimicked natural disturbance because we cleared plots of most of the vegetation before planting. By August of 2009, these coastal experimental plots were completely covered again by a dense thicket of perennial competitor species, which could limit the success of seedling recruitment in subsequent seasons and lead to an advantage for established plants. Long-term experiments could quantify any advantage gained by the inversion's effect on perenniality.

### Contribution of the Inversion Polymorphism to Reproductive Isolation

While inversions have long been thought to play a role in adaptation and formation of ecological reproductive isolation [4],[25],[55],[56], the relative contribution of inversions to these processes remains largely unknown [16]. Polymorphism for alternate arrangements of the inversion appears to be maintained between annual and perennial *M. guttatus* habitats through habitat-mediated natural selection. Thus, the inversion should have a sustained impact on multiple reproductive isolating barriers over the geographic range of the annual and perennial ecotypes.

Since the inversion only has a moderate effect on any given reproductive isolating barrier, reproductive isolation is likely to have a complex genetic architecture in this system. Further, loci affecting traits other than flowering time are already known to contribute to immigrant inviability between inland annual and coastal perennial populations. In a recent study [39], we found that coastal alleles at three salt tolerance loci are adaptive in the coastal perennial habitat but are not significantly disadvantageous in the inland annual habitat. Thus, unlike the inversion, these salt tolerance loci may only contribute to reproductive isolation in one habitat and not the other. With only a limited number of field studies conducted so far quantifying the effects of individual loci across habitats [39],[40],[57]–[59], it is unknown how commonly trade-offs at individual loci are involved in ecological reproductive isolation. However, inversions are only expected to spread by a local adaptation mechanism [12] when they capture alleles that are already near fixation as a result of fitness trade-offs across habitats. Thus, inversions spread by the local adaptation mechanism are expected to show fitness trade-offs across habitats, while genic factors in co-linear regions may not.

Inversions can directly cause postzygotic hybrid sterility as a result of the production of unbalanced gametes at meiosis in individuals heterozygous for inversion arrangements [1],[10]. No such underdominant effects on hybrid male fertility have been detected for the inversion (B. Blackman, personal communication). Multiyear studies would allow for a more comprehensive quantification of extrinsic postzygotic isolation in coastal habitat, where many plants survive beyond the first season. Given the data from our study, the strength of extrinsic isolation based on flowering versus multiseason survival should be viewed as the upper boundaries of the strength of this barrier in coastal habitat. Even so, significant extrinsic postzygotic isolation is questionable in this system as there are high levels of heterosis in the F1 generation, especially in coastal habitat [38]. Loci, such as the inversion, may be restricted in migration between habitats by extrinsic postzygotic isolation. Alleles at other loci may introgress much more easily across habitats after hybridization.

### Origins and Spread of Inversions

Phylogenetic studies have generally found that annual plant species are derived from perennial species [51],[60],[61]. Therefore, it is tempting to hypothesize that the AN arrangement, found in inland annuals and the obligate self-fertilizing annual species *M. nasutus*, is the derived chromosomal form. However, if the local adaptation mechanism [12] was responsible for the invasion of the inversion, then either arrangement would be equally likely to be derived, since capturing preexisting locally adaptive variation is the reason inversions are predicted to spread.

The *M. guttatus* species complex occurs across western North America as a mosaic of patchily distributed annual and perennial populations [38],[44],[45]. Such mosaics of divergently adapted populations with limited migration represent the ideal conditions for the invasion of inversions that capture multiple locally adapted alleles [12]. Inversions harboring multiple adaptive alleles are predicted to invade because they have a selective advantage over co-linear haplotypes, which produce descendants with unfavorable migrant alleles through recombination [12].

An alternative hypothesis is that the adaptive phenotypic effects of the inversion resulted from the inversion mutation itself causing a change in gene expression or function. Further, it is possible that adaptive mutations have accumulated within the inversion over time. Actual identification of the causative genes underlying the inversion's phenotypic effects is necessary to resolve the history of this chromosomal rearrangement and why it became so widespread. Regardless, if inversions are frequently spread by adaptations to geographically widespread divergent environmental conditions, then this could partially explain why closely related species so often differ in their chromosome structure.

## Materials and Methods

### Geographic Distribution of the Inversion

Multiple F2 mapping populations were created through crosses within and among annual and perennial ecotypes (Table S2). Tissue was collected from all F2 individuals and stored in 96-well plates at −80°C. Genomic DNA was extracted with a modified hexadecyl trimethyl-ammonium bromide chloroform extraction protocol [62].

Markers from within and on both sides of the presumed inverted region (Figure 2B) were genotyped to determine the arrangement of markers and whether or not recombination was suppressed. Primer sequences for all markers used in this study were designed previously and can be found at www.mimulusevolution.org. All PCR products were subjected to capillary electrophoresis and fragment analysis on an ABI 3730×l DNA Analyzer. The size of the amplified fragments was scored using the program GENEMARKER (SoftGenetics, 2005, State College, PA).

### Replicated QTL Analysis

Populations used in crosses for this experiment were collected in the summer of 2005 [38]. The replicated QTL analysis was conducted first with plants from the LMC/SWB and SAM/OSW population pairs in August–October 2006 followed by the CAN/BCB and RGR/OPB population pairs in March–May 2007. Finally, tests were performed on the LMC/BOG population pair in September–November 2009. The plants were grown under 18-h days at 21°C, 6-h nights at 16°C, and 30% relative humidity in 4-inch square pots at the Duke University greenhouses. Flowering time, second internode thickness and length, as well as mean corolla width and length of the first two flowers were recorded for all coastal perennial×inland annual crosses. The amount of aboveground nodes that produced roots, a trait associated with perenniality, was quantified in the 2007 experiment but not in the 2006 experiment. Only flowering time was measured in the LMC×BOG mapping population.

To be more confident that inversion effects are robust to differences in genetic background and effects were not due to rare alleles, a highly outbred breeding design was employed. For the 2006 experiment, each F2 mapping population was derived from 8–10 parental plants and involved eight different crosses to produce F1s. Due to difficulties with following multiple parental alleles in crosses in the 2006 experiment, the 2007 F2 mapping populations were only derived from four parental plants, where F1 progeny were intercrossed. This outbred design, with multiple parentals from each population, contrasts with many QTL studies where only two inbred lines are used in the initial cross. However, only one pair of parental lines was used for the LMC×BOG mapping population. For each population pair we grew 19–24 of each parental type, 17–25 F1 progeny, and 126–172 F2s. Differences in samples sizes were due to a combination of number of seeds available, germination rate, and space availability.

Multiple markers were screened in the region of the inversion. Only one marker, Micro6046, was divergent among parentals and polymorphic in all five F2 mapping populations. Micro6046 primers (F = TGATAATTTGTCCAATTGCGT, R = TCCAAATCAATAATCAAATCCC) were designed using Primer3 (rodo.wi.mit.edu/primer3/) targeted to a microsatellite on a sequenced bacterial artificial chromosome (GenBank accession number 154350257), which was incorporated as part scaffold 11 of the *M. guttatus* genome assembly v1.0, within the known inverted region (www.phytozome.net).

We tested for an association between Micro6046 and a composite of five traits (flowering time, internode thickness, internode length, corolla width, and corolla length) with a MANOVA for each population pair. To test for an association between the Micro6046 and individual traits, we conducted separate one-way ANOVAs. All analyses were carried out in JMP 7.0.1 (SAS, Cary, NC).

### Creation of Backcross Introgression Lines

Crosses between three independent sets of LMC (inland annual) and SWB (coastal perennial) inbred lines were used to initiate backcross introgression line breeding (Figure 3). These populations were selected because they had been successfully used in a previous reciprocal transplant field experiment [38]. This led to the production of three independently derived F1 progeny, which were then reciprocally backcrossed as the pollen donor to the parental lines from which they were derived. Parental lines were also self-fertilized each generation. Thus, all lines became progressively more inbred each generation.

To facilitate the introgression of the inversion into each genetic background, two flanking makers (e571, e772) were genotyped outside of the inversion and one marker (e173) was genotyped in the middle of the inversion. Each generation, 32 backcross individuals of each type were genotyped for these markers. Individuals heterozygous for the three markers were then backcrossed to each inbred parental line. Fourth generation backcrosses were then self-fertilized and progeny were used for crosses to create the final generation. In the penultimate generation, plants homozygous for either the AN or PE arrangements were selected through genotyping to create the final generation. To eliminate effects of inbreeding depression, the final generation of breeding involved intercrosses within independently derived lines of the same genetic background and introgression type (*N* = 3 backcross lines with introgressed region and 3 backcross lines where introgression was selected against in last generation per two genetic backgrounds = 12 crosses; see Figure 3). Intercrosses were also conducted among inbred parental lines to create outbred lines for the reciprocal transplant experiment.

### Field Quantification of Inversion Polymorphism Effects

Seeds from all outbred lines (Figure 3) were sown in plug trays filled with Ocean Forest Potting Soil (Fox Farm, Arcata, CA) on February 8, 2009 in the Bodega Marine Reserve greenhouse. Germination was achieved on a regime of misting three times daily for 5 min with no supplemental lighting.

One inland annual (Boonville, CA N 38°59.221, W 123°21.059, Figure S4) and one coastal perennial (Manchester, CA N 39°00.498, W 123°41.637, Figure S5) field site [38] were selected for the reciprocal transplant experiment. The experimental design included 20 blocks per site with 10 replicates of each backcross line randomized within blocks. Due to low germination success of some lines, we were not able to achieve 10 replicates in all blocks. To prevent trampling by livestock and local deer populations, exclosures were set up around the blocks. Transplantation of seedlings to the field sites was conducted from March 9–16, 2009.

Field sites were censused over the course of the experiment at different intervals based on results from a previous field experiment [38]. Survivorship, flowering time, and number of flowers produced were recorded during each census. Data were collected at the inland site until June 23, 2009, when all plants had died as a result of the summer drought. At the coastal site, data collection was terminated on October 29, 2009 because flowering had ceased and plots were overgrown with other plant species. For the final coastal census, all remaining plants were carefully removed from the blocks, survivorship was assessed, and the final number of flowers produced was derived from counts of fruits.

### Analysis of Field Data

To determine whether the inversion contributed to fitness effects across field sites, data were analyzed with ASTER [63],[64], which is a module of the statistical program R (R Core Development Team 2009). ASTER modeling allows for a single analysis of multiple components of fitness, while correctly accounting for their order of occurrence and different probability distributions. ASTER accounts for dependencies among fitness components by generating an overall likelihood for each individual over the course of its life. ASTER was used here to analyze the composite of two fitness components: Survival to flowering, modeled as Bernoulli (0 or 1), and the number of flowers per surviving individual, which was modeled as a zero-truncated Poisson distribution. Initially, the parents of the backcross lines were analyzed alone to test effects of site, genotype, and site×genotype interaction. Nested null models were used for comparison to test these alternative hypotheses through likelihood ratio tests.

To determine the effect of the inversion across habitats, a similar analysis was conducted on the same two components of fitness as the parents but this time using the data from the backcross lines. The effects of site, genetic background, inversion allele, and all the interactions of these three factors were tested by fitting a series of nested models and comparing them with likelihood ratio tests. To test the significance of any given factor, null models were compared to alternative models that only differed by the addition of the factor of interest. Finally, maximum likelihood estimates of fitness were calculated with ASTER for all parental and backcross lines across both field sites.

To test for effects of genetic background and the inversion on survival over the course of the season, survival analysis was conducted using ASTER, where survivorship for each field census was modeled as Bernoulli. The dates of censuses as well as the total number of censuses differed between the coastal (*N* = 11) and inland (*N* = 16) field sites. Therefore, separate analyses were conducted for each field site to test the effects of the inversion on survival.

To determine if there was an effect of the inversion on flowering time divergence, two-way ANOVAs in JMP 7.0.1 (SAS, Cary, NC) were conducted within each field site, with the inversion and genetic background as factors. The least square means for inversion alleles were used as a quantification of the magnitude of its effect on flowering time.

### Quantification of Reproductive Isolating Barrier Strengths

The overall strength of ecological reproductive isolating barriers was quantified using previously developed methods [13],[46]. To calculate the effect of the inversion locus on any given barrier, we used the general formula *RI_Inversion_* = *RI_Foreign_*−*RI_Native_*, where *RI_Foreign_* is reproductive isolation between the native population and the backcross line with the foreign inversion arrangement and *RI_Native_* is reproductive isolation between the native population and the backcross line with the native inversion arrangement.

Temporal flowering time isolation between habitats was calculated from the flowering distribution of LMC plants at the inland field site and SWB plants at the coastal field site, with calculations made relative to each other. All quantifications of temporal isolation were calculated for each native population (LMC or SWB) as the pollen recipient relative to a foreign pollen donor at the foreign field site. The individual contribution of the inversion to between habitat temporal isolation was calculated as *RI_TBH,Inversion_* = *RI_TBH,f,f,f_*−*RI_TBH,n,f,f_*, where *RI_TBH,f,f,f_* is the temporal reproductive isolation of the native parent relative to a pollen donor backcross line with the foreign arrangement in the foreign genetic background at the foreign field site, and *RI_TBH,n,f,f_* is the temporal reproductive isolation of the native parent relative to the backcross line with the native arrangement in the foreign genetic background at the foreign field site.

Reproductive isolation due to selection against immigrants was quantified as 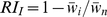, where 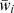 is the expected number of flowers produced (ASTER predicted) by foreign individuals, and 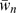 is the expected number of flowers produced by native individuals. The contribution of the inversion to reproductive isolation through selection against immigrants was calculated as: 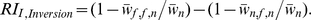



Here, 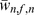 is the expected number of flowers produced by the backcross line with the native arrangement in the foreign genetic background at the native field site and 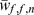 is the expected number of flowers produced by the backcross line with the foreign arrangement in the foreign genetic background at the native field site.

Temporal flowering time reproductive isolation in sympatry was calculated for the native parental (SWB or LMC) relative to the foreign parental within each field site. The contribution of the inversion was calculated as *RI_TS,Inversion_* = *RI_TS,f,f,n_*−*RI_TS,n,f,n_*, where *RI_TS,f,f,n_* is the temporal reproductive isolation in sympatry of the native parent relative to a pollen donor with the foreign arrangement in the foreign genetic background at the native field site, and *RI_TBH,n,f,n_* is the temporal reproductive isolation of the native parent relative to a pollen donor with the native arrangement in the foreign genetic background at the native field site.

The strength of extrinsic postzygotic reproductive isolation was calculated with data from a previous reciprocal transplant experiment that incorporated F1 progeny [38] as 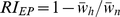. Here, 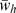 is the fitness of F1 progeny in the field, and 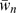 is the fitness of native parental plants at each field site. Extrinsic postzygotic isolation for the inversion locus was calculated at each site as 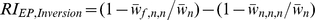 with the data from this study. Here, 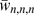 is the fitness of the backcross line with the native arrangement in the native genetic background at the native field site and 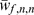 is the fitness of the backcross lines with the foreign arrangement in the native genetic background at the native field site. Because of multi-season survival at the coastal site, extrinsic postzygotic isolation was calculated for two different components of fitness: expected number of flowers in the first season and survival to the second season.

## Supporting Information

Figure S1 Annual rainfall and temperatures. Thirty year (1961–1990) average monthly data from the closest weather stations (Ukiah: Inland, Point Arena: Coast) to the field sites of the reciprocal transplant experiments. (A) Rainfall (mm) in coast (blue) and inland (orange) habitats. (B) Average high (coast: blue, inland: orange) and low (coast: purple, inland: red) temperatures.
(0.70 MB TIF)

Figure S2 Histogram of F2 flowering time. Distribution of days to first flower under greenhouse conditions for progeny of crosses between (A) CAN and BCB, (B) LMC and SWB, (C) RGR and OPB, and (D) SAM and OSW. Orange and blue arrows indicate the mean flowering time for the inland annual and coastal perennial parental types, respectively.
(0.55 MB TIF)

Figure S3 Histogram of F2 stem thickness. Distribution of second internode thickness under greenhouse conditions for progeny of crosses between (A) CAN and BCB, (B) LMC and SWB, (C) RGR and OPB, and (D) SAM and OSW. Orange and blue arrows indicate the mean stem thickness for the inland annual and coastal perennial parental types, respectively.
(0.39 MB TIF)

Figure S4 Photos documenting onset of drought at inland field site. View of the inland annual field site (Boonville, CA) from same perspective over the course of the spring on (A) March 3, (B) May 7, and (C) June 12, 2009.
(3.65 MB TIF)

Figure S5 Photo of the coastal perennial field site. Located near Manchester, CA in a seep on a cliff at the edge of a coastal terrace formation.
(5.73 MB TIF)

Table S1 Geographic locations of populations used in this study.
(0.02 MB XLS)

Table S2 Geographic distribution of chromosomal inversion arrangement as determined by crosses.
(0.02 MB XLS)

Table S3 Morphological trait variation in crosses between ecotypes measured in the greenhouse.
(0.02 MB XLS)

Table S4 Fitness analysis of coastal perennial and inland annual parental ecotypes from the field experiment with ASTER.
(0.02 MB XLS)

## References

[1] Stebbins G. L (1958). The inviability, weakness, and sterility of interspecific hybrids.. Adv Genet.

[2] Mayr E (1982). Speciation and macroevolution.. Evolution.

[3] Clausen J (1951). Stages in the evolution of plant species.

[4] Dobzhansky T (1970). Genetics of the evolutionary process.

[5] White M. J. D (1978). Modes of speciation.

[6] King M (1993). Species evolution: the role of chromosomal change.

[7] Rieseberg L. H (2001). Chromosomal rearrangements and speciation.. Trends Ecol Evol.

[8] Noor M. A. F, Grams K. L, Bertucci L. A, Reiland J (2001). Chromosomal inversions and the reproductive isolation of species.. P Natl Acad Sci U S A.

[9] Navarro A, Barton N. H (2003). Chromosomal speciation and molecular divergence: accelerated evolution in rearranged chromosomes.. Science.

[10] Coyne J. A, Orr H. A (2004). Speciation.

[11] Butlin R. K (2005). Recombination and speciation.. Mol Ecol.

[12] Kirkpatrick M, Barton N (2006). Chromosome inversions, local adaptation and speciation.. Genetics.

[13] Lowry D. B, Modliszewski J. L, Wright K. M, Wu C. A, Willis J. H (2008). The strength and genetic basis of reproductive isolating barriers in flowering plants.. Philos T R Soc B.

[14] Noor M. A. F, Bennett S. M (2009). Islands of speciation or mirages in the desert? Examining the role of restricted recombination in maintaining species.. Heredity.

[15] Lai Z, Nakazato T, Salmaso M, Burke J. M, Tang S. X (2005). Extensive chromosomal repatterning and the evolution of sterility barriers in hybrid sunflower species.. Genetics.

[16] Hoffmann A. A, Rieseberg L. H (2008). Revisiting the impact of inversions in evolution: from population genetic markers to drivers of adaptive shifts and speciation?. Annu Rev Ecol Evol S.

[17] Ortiz-Barrientos D, Reiland J, Hey J, Noor M. A. F (2002). Recombination and the divergence of hybridizing species.. Genetica.

[18] Ortiz-Barrientos D, Counterman B. A, Noor M. A. F (2004). The genetics of speciation by reinforcement.. PLoS Biol.

[19] Felsenstein J (1981). Skepticism toward Santa Rosalia, or why are there so few kinds of animals.. Evolution.

[20] Trickett A. J, Butlin R. K (1994). Recombination suppressors and the evolution of new species.. Heredity.

[21] Servedio M. R (2000). Reinforcement and the genetics of nonrandom mating.. Evolution.

[22] Linhart Y. B, Grant M. C (1996). Evolutionary significance of local genetic differentiation in plants.. Annu Rev Ecol Evol S.

[23] Kawecki T. J, Ebert D (2004). Conceptual issues in local adaptation.. Ecol Lett.

[24] Dobzhansky T (1951). Genetics and the origin of species (3rd ed.).

[25] Lewis H (1953). The mechanism of evolution in the genus *Clarkia*.. Evolution.

[26] Rodriguez-Trelles F, Rodriguez M. A (1998). Rapid micro-evolution and loss of chromosomal diversity in *Drosophila* in response to climate warming.. Evol Ecol.

[27] Feder J. L, Xie X. F, Rull J, Velez S, Forbes A (2005). Mayr, Dobzhansky, and Bush and the complexities of sympatric speciation in *Rhagoletis*.. P Natl Acad Sci U S A.

[28] Umina P. A, Weeks A. R, Kearney M. R, McKechnie S. W, Hoffmann A. A (2005). A rapid shift in a classic clinal pattern in *Drosophila* reflecting climate change.. Science.

[29] Balanya J, Oller J. M, Huey R. B, Gilchrist G. W, Serra L (2006). Global genetic change tracks global climate warming in *Drosophila subobscura*.. Science.

[30] Etges W. J, Levitan M (2004). Palaeoclimatic variation, adaptation and biogeography of inversion polymorphisms in natural populations of *Drosophila robusta*.. Biol J Linn Soc.

[31] Dubinin N. P, Tiniakov G. G (1945). Seasonal cycles and the concentration of inversions in populations of *Drosophilia funebris*.. Am Nat.

[32] Dubinin N. P, Tiniakov G. G (1946). Seasonal cycle and inversion frequency in populations.. Nature.

[33] Stalker H. D, Carson H. L (1949). Seasonal variation in the morphology of *Drosophila robusta* Sturtevant.. Evolution.

[34] Feder J. L, Roethele F. B, Filchak K, Niedbalski J, Romero-Severson J (2003). Evidence for inversion polymorphism related to sympatric host race formation in the apple maggot fly, *Rhagoletis pomonella*.. Genetics.

[35] Feder J. L, Berlocher S. H, Roethele J. B, Dambroski H, Smith J. J (2003). Allopatric genetic origins for sympatric host-plant shifts and race formation in *Rhagoletis*.. P Natl Acad Sci U S A.

[36] Wu C. A, Lowry D. B, Cooley A. M, Wright K. M, Lee Y. W, Willis J. H (2008). *Mimulus* is an emerging model system for the integration of ecological and genomic studies.. Heredity.

[37] Hall M. C, Willis J. H (2006). Divergent selection on flowering time contributes to local adaptation in *Mimulus guttatus* populations.. Evolution.

[38] Lowry D. B, Rockwood R. C, Willis J. H (2008). Ecological reproductive isolation of coast and inland races of *Mimulus guttatus*.. Evolution.

[39] Lowry D. B, Hall M. C, Salt D. E, Willis J. H (2009). Genetic and physiological basis of adaptive salt tolerance divergence between coastal and inland *Mimulus guttatus*.. New Phytol.

[40] Hall M. C, Lowry D. B, Willis J. H (2010). Is local adaptation in *Mimulus guttatus* caused by trade-offs at individual loci?. Mol Ecol.

[41] Corbin J. D, Thomsen M. A, Dawson T. E, D'Antonio C. M (2005). Summer water use by California coastal prairie grasses: fog, drought, and community composition.. Oecologia.

[42] Hall M. C, Basten C. J, Willis J. H (2006). Pleiotropic quantitative trait loci contribute to population divergence in traits associated with life-history variation in *Mimulus guttatus*.. Genetics.

[43] Sweigart A. L, Willis J. H (2003). Patterns of nucleotide diversity in two species of *Mimulus* are affected by mating system and asymmetric introgression.. Evolution.

[44] Clausen J, Hiesey W. M (1958). Experimental studies on the nature of species. IV. Genetic structure of ecotypes.

[45] van Kleunen M (2007). Adaptive genetic differentiation in life-history traits between populations of *Mimulus guttatus* with annual and perennial life-cycles.. Evol Ecol.

[46] Martin N. H, Willis J. H (2007). Ecological divergence associated with mating system causes nearly complete reproductive isolation between sympatric *Mimulus* species.. Evolution.

[47] Manoukis N. C, Powell J. R, Toure M. B, Sacko A, Edillo F. E (2008). A test of the chromosomal theory of ecotypic speciation in *Anopheles gambiae*.. P Natl Acad Sci U S A.

[48] McAllister B. F, Sheeley S. L, Mena P. A, Evans A. L, Schlotterer C (2008). Clinal distribution of a chromosomal rearrangement: a precursor to chromosomal speciation?. Evolution.

[49] Silvertown J. W, Charlesworth D (2001). Introduction to plant population biology.

[50] Roux F, Touzet P, Cuguen J, Le Corre V (2006). How to be early flowering: an evolutionary perspective.. Trends Plant Sci.

[51] Datson P. M, Murray B. G, Steiner K. E (2008). Climate and the evolution of annual/perennial life-histories in *Nemesia* (Scrophulariaceae).. Plant Syst Evol.

[52] McKay J. K, Richards J. H, Nemali K. S, Sen S, Mitchell-Olds T (2008). Genetics of drought adaptation in *Arabidopsis thaliana* II. QTL analysis of a new mapping population, KAS-1×TSU-1.. Evolution.

[53] Wu C. A, Lowry D. B, Nutter L. I, Willis J. H (2010). Natural variation for drought-response traits in the *Mimulus guttatus* species complex.. Oecologia.

[54] Hampton M. A, Griggs G. B (2004). Formation, evolution, and stability of coastal cliffs-status and trends..

[55] Grant V (1981). Plant speciation, 2nd ed.

[56] Gottlieb L. D (2004). Rethinking classic examples of recent speciation in plants.. New Phytol.

[57] Verhoeven K. J. F, Poorter H, Nevo E, Biere A (2008). Habitat-specific natural selection at a flowering-time QTL is a main driver of local adaptation in two wild barley populations.. Mol Ecol.

[58] Verhoeven K. J. F, Vanhala T. K, Biere A, Nevo E, Van Damme J. M. M (2004). The genetic basis of adaptive population differentiation: a quantitative trait locus analysis of fitness traits in two wild barley populations from contrasting habitats.. Evolution.

[59] Gardner K. M, Latta R. G (2006). Identifying loci under selection across contrasting environments in *Avena barbata* using quantitative trait locus mapping.. Mol Ecol.

[60] Andreasen K, Baldwin B. G (2001). Unequal evolutionary rates between annual and perennial lineages of checker mallows (*Sidalcea*, Malvaceae): evidence from 18S-26S rDNA internal and external transcribed spacers.. Mol Biol Evol.

[61] Church S. A (2003). Molecular phylogenetics of *Houstonia* (Rubiaceae): descending aneuploidy and breeding system evolution in the radiation of the lineage across North America.. Mol Phylogenet Evol.

[62] Kelly A. J, Willis J. H (1998). Polymorphic microsatellite loci in *Mimulus guttatus* and related species.. Mol Ecol.

[63] Geyer C. J, Wagenius S, Shaw R. G (2007). Aster models for life history analysis.. Biometrika.

[64] Shaw R. G, Geyer C. J, Wagenius S, Hangelbroek H. H, Etterson J. R (2008). Unifying life-history analyses for inference of fitness and population growth.. Am Nat.

