# Correction: A Widespread Chromosomal Inversion Polymorphism Contributes to a Major Life-History Transition, Local Adaptation, and Reproductive Isolation

**DOI:** 10.1371/annotation/caa1b7dd-9b6d-44db-b6ce-666954903625

**Published:** 2012-01-09

**Authors:** David B. Lowry, John H. Willis

There is an error in the legend for Figure 4, “Effects of the inversion locus across field sites in different genetic backgrounds” panels A-F.

The complete, correct Figure 4 legend is: “(A) Proportion of plants surviving to flower and (B) expected fitness produced per plants across field sites. Values plotted are maximum likelihood estimates ±1 SE calculated with ASTER. (C) Cumulative proportion of plants surviving to flower and (D) expected fitness per individual at the inland field site. Survival over time at the (E) coastal perennial and (F) inland annual field sites. Parental lines: yellow, inland annual parent; blue, coastal perennial parent. Backcross lines: red, PE arrangement in annual genetic background; orange, AN arrangement in annual genetic background; green, AN arrangement in perennial genetic background; pink, PE arrangement in perennial genetic background.” 

