# Supplementary figures and images for: A Widespread Chromosomal Inversion Polymorphism Contributes to a Major Life-History Transition, Local Adaptation, and Reproductive Isolation
Source: PLoS Biol. 2010 Sep 28;8(9):e1000500. doi: 10.1371/journal.pbio.1000500 (PMC2946948; doi:10.1371/journal.pbio.1000500)

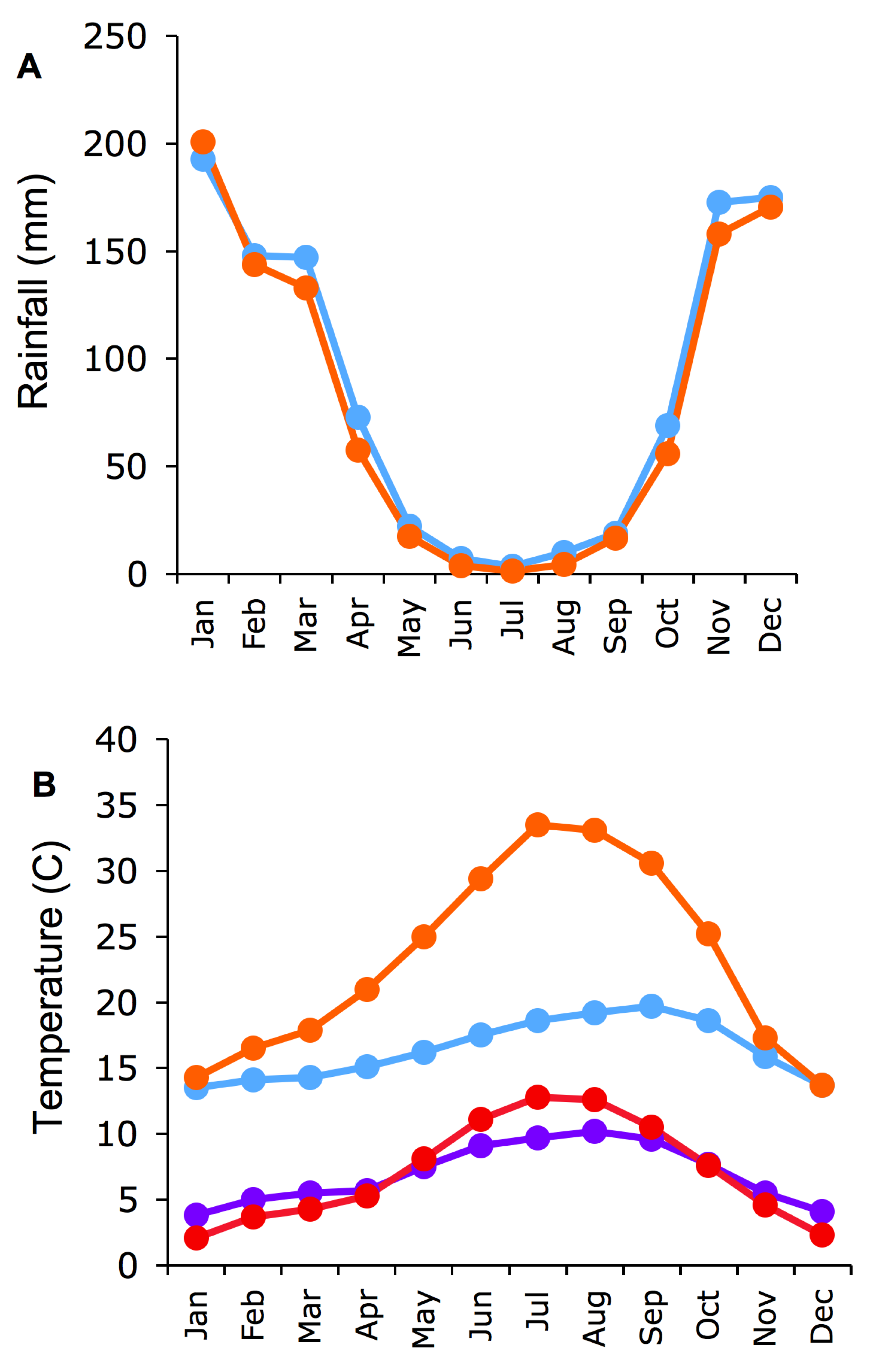

Supplement: Figure S1 — Annual rainfall and temperatures. Thirty year (1961–1990) average monthly data from the closest weather stations (Ukiah: Inland, Point Arena: Coast) to the field sites of the reciprocal transplant experiments. (A) Rainfall (mm) in coast (blue) and inland (orange) habitats. (B) Average high (coast: blue, inland: orange) and low (coast: purple, inland: red) temperatures. (0.70 MB TIF) [file pbio.1000500.s001.tif]

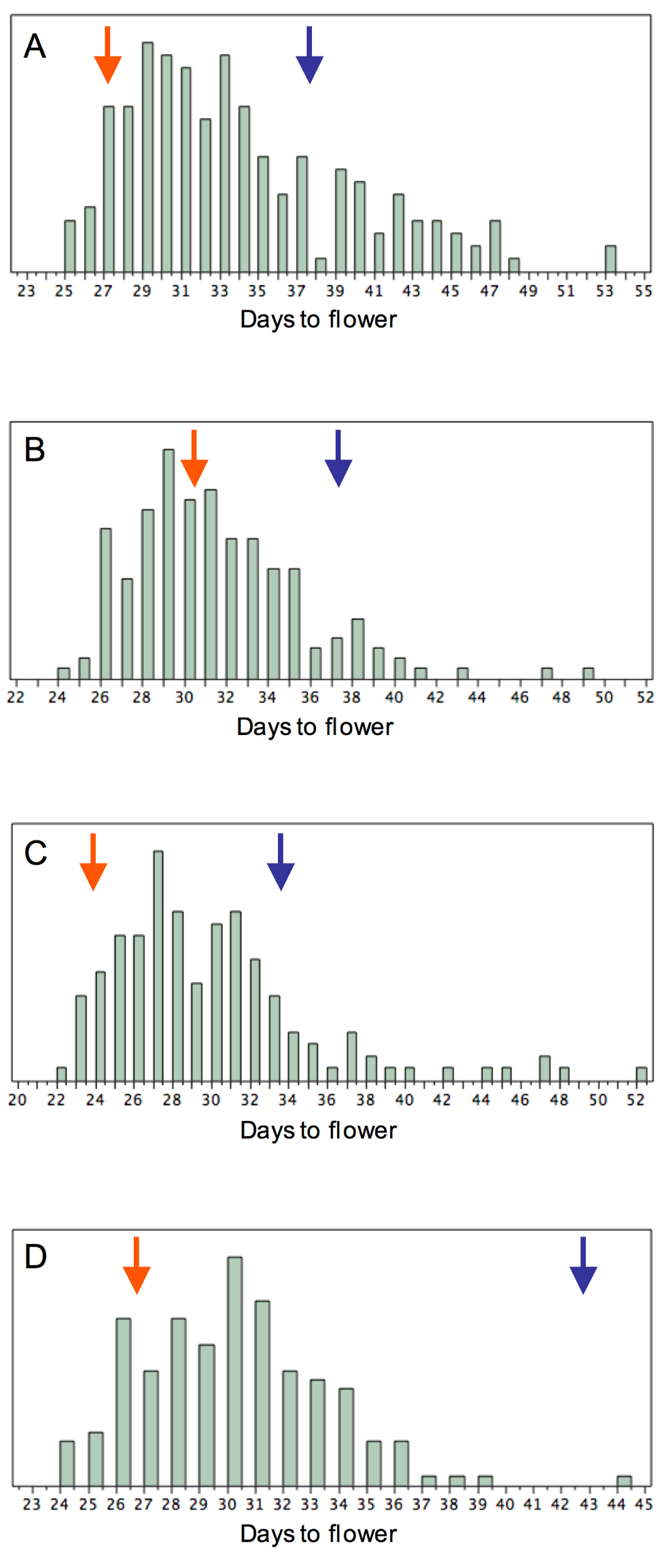

Supplement: Figure S2 — Histogram of F2 flowering time. Distribution of days to first flower under greenhouse conditions for progeny of crosses between (A) CAN and BCB, (B) LMC and SWB, (C) RGR and OPB, and (D) SAM and OSW. Orange and blue arrows indicate the mean flowering time for the inland annual and coastal perennial parental types, respectively. (0.55 MB TIF) [file pbio.1000500.s002.tif]

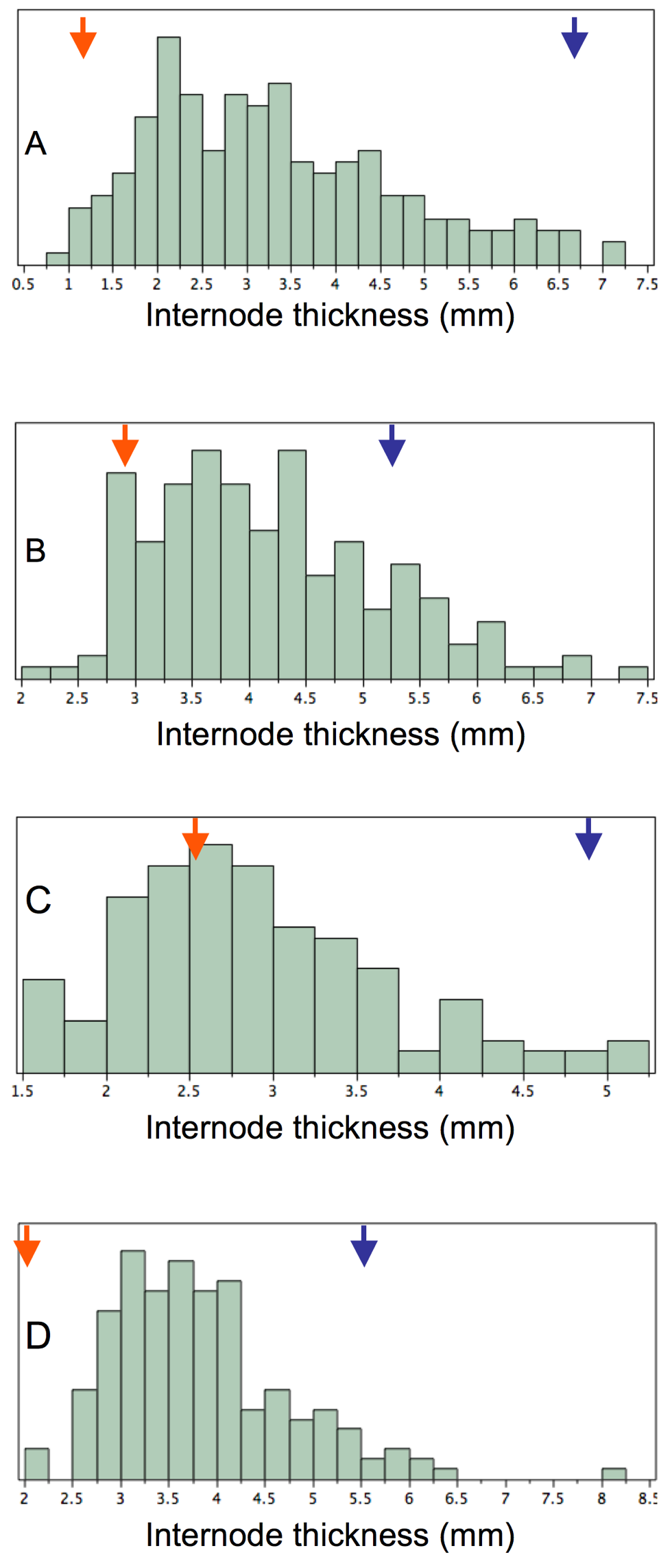

Supplement: Figure S3 — Histogram of F2 stem thickness. Distribution of second internode thickness under greenhouse conditions for progeny of crosses between (A) CAN and BCB, (B) LMC and SWB, (C) RGR and OPB, and (D) SAM and OSW. Orange and blue arrows indicate the mean stem thickness for the inland annual and coastal perennial parental types, respectively. (0.39 MB TIF) [file pbio.1000500.s003.tif]

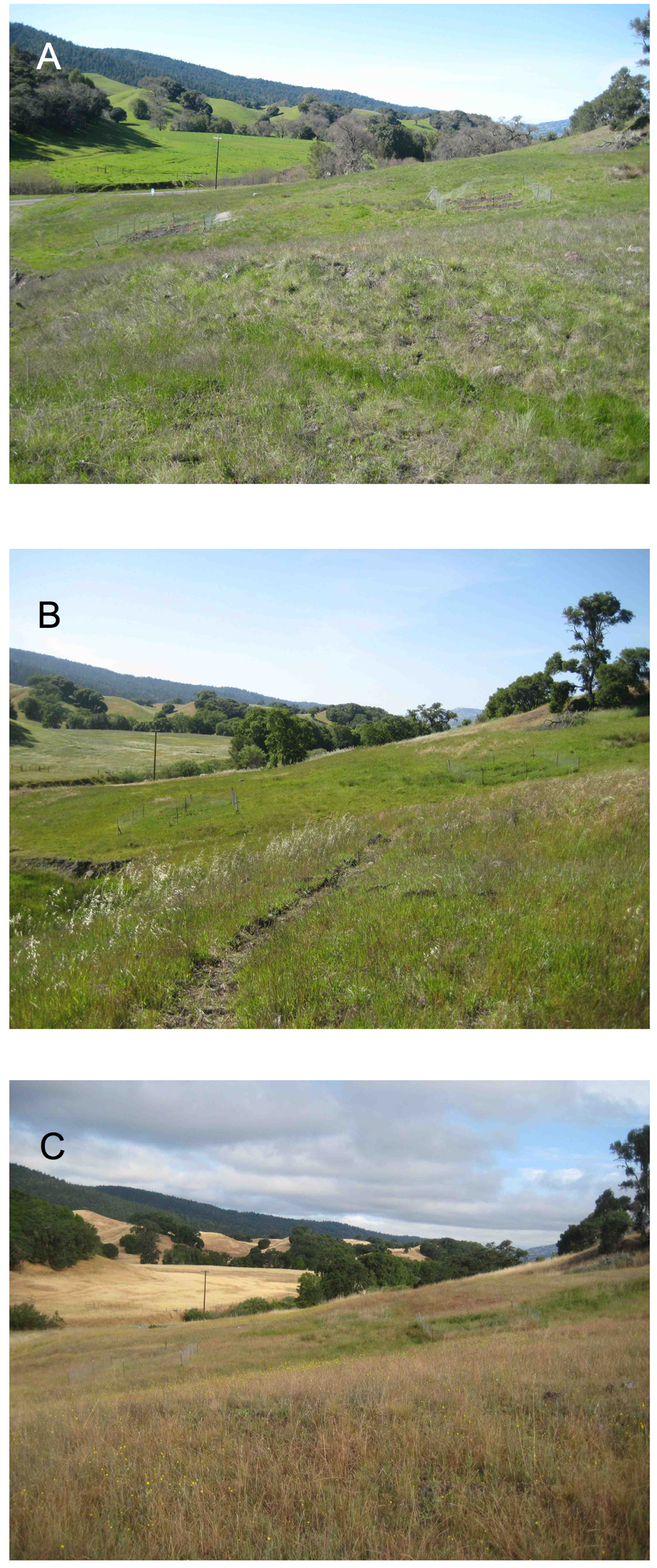

Supplement: Figure S4 — Photos documenting onset of drought at inland field site. View of the inland annual field site (Boonville, CA) from same perspective over the course of the spring on (A) March 3, (B) May 7, and (C) June 12, 2009. (3.65 MB TIF) [file pbio.1000500.s004.tif]

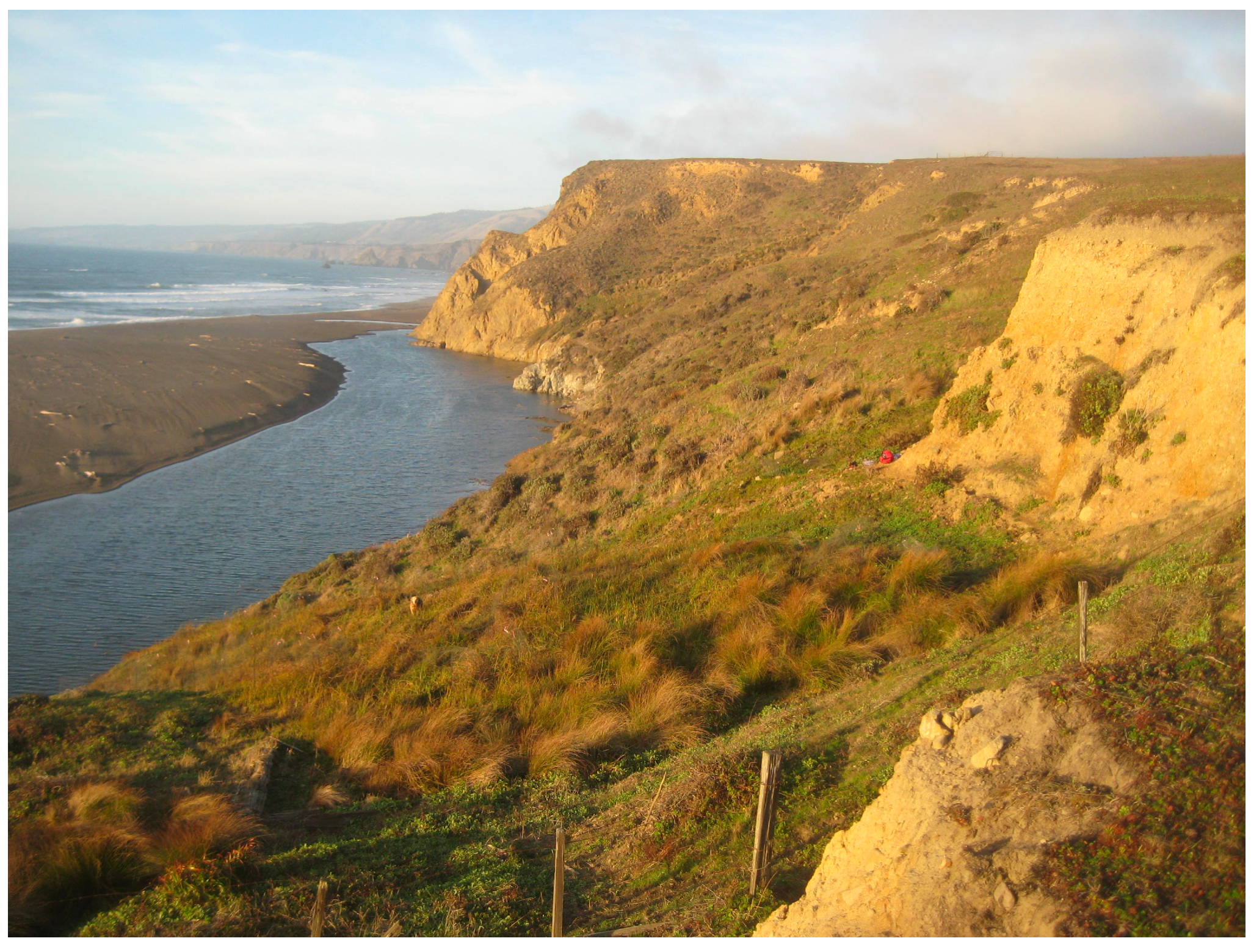

Supplement: Figure S5 — Photo of the coastal perennial field site. Located near Manchester, CA in a seep on a cliff at the edge of a coastal terrace formation. (5.73 MB TIF) [file pbio.1000500.s005.tif]
